# Glucose-6-Phosphate Acts as an Extracellular Signal of SagS To Modulate *Pseudomonas aeruginosa* c-di-GMP Levels, Attachment, and Biofilm Formation

**DOI:** 10.1128/mSphere.01231-20

**Published:** 2021-02-10

**Authors:** Soyoung Park, Jozef Dingemans, Madison Gowett, Karin Sauer

**Affiliations:** a Department of Biological Sciences, Binghamton University, Binghamton, New York, USA; b Binghamton Biofilm Research Center, Binghamton University, Binghamton, New York, USA; University of Georgia

**Keywords:** *P. aeruginosa*, SagS, glucose-6-phosphate, phosphorylation, fructose-1,6-bisphosphate, β-methyl-d-galactoside, biofilm, Biolog, NicD, c-di-GMP, interactome, pulldown

## Abstract

In Pseudomonas aeruginosa, the orphan two-component sensor SagS contributes both to transition to biofilm formation and to biofilm cells gaining their heightened tolerance to antimicrobials. However, little is known about the identity of the signals or conditions sensed by SagS to induce the switch to the sessile, drug-tolerant mode of growth. Using a modified Biolog phenotype assay to screen for compounds that modulate attachment in a SagS-dependent manner, we identified glucose-6-phosphate to enhance attachment in a manner dependent on the glucose-6-phosphate concentration and SagS. The stimulatory effect was not limited to the attachment since glucose-6-phosphate likewise enhanced biofilm formation and also enhanced the expression of select biofilm marker genes. Moreover, exposure to glucose-6-phosphate coincided with decreased swarming motility but increased cellular cyclic-di-GMP (c-di-GMP) levels in biofilms. No such response was noted for compounds modulating attachment and biofilm formation in a manner independent of SagS. Modulation of c-di-GMP in response to glucose-6-phosphate was due to the diguanylate cyclase NicD, with NicD also being required for enhanced biofilm formation. The latter was independent of the sensory domain of NicD but dependent on NicD activity, SagS, and the interaction between NicD and SagS. Our findings indicate that glucose-6-phosphate likely mimics a signal or conditions sensed by SagS to activate its motile-sessile switch function. In addition, our findings provide new insight into the interfaces between the ligand-mediated two-component system signaling pathway and c-di-GMP levels.

**IMPORTANCE** Pathogens sense and respond to signals and cues present in their environment, including host-derived small molecules to modulate the expression of their virulence repertoire. Here, we demonstrate that the opportunistic pathogen Pseudomonas aeruginosa responds to glucose-6-phosphate. Since glucose-6-phosphate is primarily made available due to cell lysis, it is likely that glucose-6-phosphate represents a cross-kingdom cell-to-cell signal that enables P. aeruginosa to adapt to the (nutrient-poor) host environment by enhancing biofilm formation, cyclic-di-GMP, and the expression of genes linked to biofilm formation in a concentration- and SagS-dependent manner.

## INTRODUCTION

To adapt and survive in a wide range of niches, bacteria are capable of sensing and responding to diverse environmental changes. Such adaptations in turn alter cellular physiological processes such as cell cycle control, cell wall homeostasis, and ion transport, as well as a change in the mode of growth, including biofilm formation (1–5). Biofilms are multicellular communities encased in a self-produced polymeric matrix and attached to the surface (6). The formation of biofilms is initiated upon surface contact and subsequently proceeds through a number of distinct stages, including irreversible attachment, stage 1 maturation, stage 2 maturation, and lastly, dispersion that enables bacteria to reinitiate the formation of biofilms at other locales (7).

These cellular changes coinciding with the transition from the planktonic to the biofilm mode of growth in response to physiological changes are driven by complex regulatory networks that not only contribute to the differential production of appendages (flagella and type IV pili) or the biofilm polymeric matrix that impact biofilm development (8–10) but also function to perceive and integrate environmental cues into signaling relays. Environmental cues are predominantly transduced into the bacterial cell by two-component regulatory systems (TCS) (11, 12). These systems generally consist of a receptor histidine kinase that recognizes a specific cue(s) or signal(s) and modifies the activity of a cognate response regulator through phosphorylation, which in turn modulates the expression of a subset of gene and subsequently cellular physiological processes (13). For example, the KinD sensor kinase by Bacillus subtilis enhances biofilm formation in response to the combination of glycerol and manganese ions (Mn^2+^) (14), while the B. subtilis sensor kinase, KinB, recognizes low oxygen levels by interacting with the respiratory apparatus and thereby stimulates matrix production and the consequent colony wrinkling (15). In Pseudomonas aeruginosa, the Gac/Rsm pathway-associated hybrid histidine kinase, LadS, binds to calcium ions to activate the TCS GacS/GacA, which in turn promotes the transcription of two small regulatory RNAs (sRNAs), RsmY and RsmZ (4). These sRNAs sequester RsmA, a repressor that hinders genes involved in biofilm formation (16). Signaling relay via TCS is not limited to extracellular cues, as evidenced by Escherichia coli, for which sensing of the intercellular signal indole has been linked to the CpxA sensor kinase and its cognate response regulator CpxR, with indole sensing coinciding with the repression of flagellar genes and the modulation of biofilm formation (17, 18).

The transition from the planktonic lifestyle to the biofilm mode of growth is regulated by multiple overlapping signaling systems. In the opportunistic Gram-negative pathogen P. aeruginosa, for example, there are multiple overlapping signaling systems such as the Gac/Rsm TCS pathway, that fine-tune alterations in cyclic-di-GMP (c-di-GMP) levels, motility, and surface attachment (19–22). One key player in the biofilm regulation networks that has been linked to planktonic-specific Gac/Rsm-dependent signaling and c-di-GMP regulation is the sensor-regulator hybrid SagS (23, 24). SagS was previously identified to interact with and phosphorylate the GacA-dependent histidine phosphotransfer protein HptB (23). Under planktonic conditions, SagS modulates the levels of sRNA, which are core components of the Gac/Rsm pathway, in an HptB-dependent manner (24, 25). When HptB is dephosphorylated, HsbR kinase phosphorylates the anti-anti-sigma factor HsbA, with phosphorylated HsbA, which in turn interacts with the diguanylate cyclase HsbD (22). Interaction with HsbD consequently leads to increased levels of c-di-GMP and increased abundance of the small rRNA RsmY (22). Under biofilm growth conditions, SagS independently facilitates biofilm formation and the activation of biofilm-associated antimicrobial tolerance (24, 26). More specifically, SagS promotes the biofilm formation via hierarchical phosphotransfer-based signaling to the BfiSR two-component regulatory system, which in turn contributes to the regulation of the sRNA, RsmZ, levels (27, 28). In addition, SagS regulates the switch from an antimicrobial susceptible to a highly tolerant state via regulation of c-di-GMP levels and subsequent activation of BrlR, a c-di-GMP responsive transcriptional regulator of biofilm resistance, in a manner independent of phosphotransfer. BrlR, in turn, activates the expression of multidrug efflux pumps and ABC transporter (26, 29, 30).

SagS is a transmembrane protein consisting of an N-terminal HmsP domain, a histidine kinase (HisKA), and a C-terminal response regulator receiver (Rec) domain (31). The periplasmic HmsP domain of SagS is predicted to perceive specific signals or cues and acts as a control point in the regulation of biofilm formation and biofilm tolerance (32, 33). However, how SagS is activated and how it stimulates c-di-GMP levels and two different phenotypic switches is not yet fully understood. More specifically, environmental cue(s) that activate SagS to modulate the two phenotypic switches still remain unknown.

Here, we performed a modified Biolog phenotype assay to explore compound(s) that activate SagS, by first screening for compounds that modulate attachment in a SagS-dependent manner. By doing so, we found glucose-6-phosphate to enhance attachment and biofilm formation, with the stimulatory effect being dependent SagS. We further demonstrate that exposure to glucose-6-phosphate is a prerequisite for elevated of c-di-GMP levels in biofilms and that modulation of the c-di-GMP pool is linked to the diguanylate cyclase NicD and its interaction with SagS. Therefore, our findings provide new insight into the interplay between TCS and c-di-GMP signaling.

## RESULTS

### Identification of compounds capable of modulating attachment in a SagS-dependent manner.

Two-component hybrid SagS enables the transition from initial attachment to the biofilm developmental progression and recalcitrance of biofilm cells to antimicrobial agents via distinct regulatory circuits (31). Moreover, we recently demonstrated that the periplasmic sensory HmsP domain of SagS acts as a control point which promotes its dual function (32). However, the mechanism by which SagS is activated to induce the switch remains to be elucidated. Considering that several amino acid residues essential for SagS function have previously been reported to locate to within a fold of beta-sheet region similar to a binding pocket (32), which in other sensing domains has been identified as a site of ligand binding, we hypothesized that SagS interacts with a small molecule. Given that SagS activates attachment and subsequent biofilm formation (24), we further reasoned that exposure to such a compound would modulate both attachment and biofilm formation by P. aeruginosa wild type but not by a P. aeruginosa strain lacking SagS.

**FIG 1:**
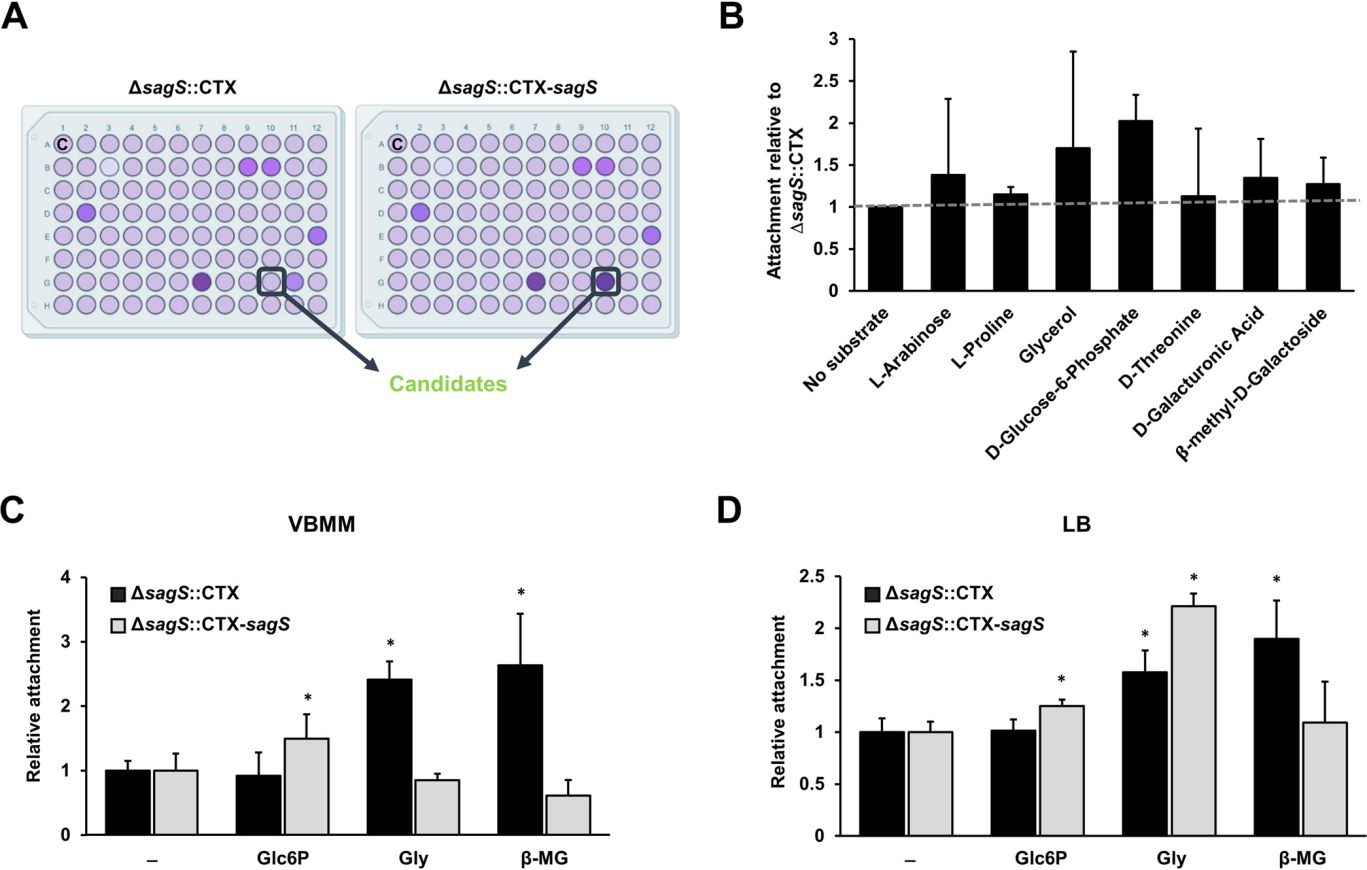
Identification of compounds that enhance attachment in a SagS-dependent manner. (A) A high-throughput screening assay was used to identify potential compounds that activate SagS. The screening was performed using phenotyping microarray (PM) plates (Biolog, Inc.) containing various carbon and nitrogen sources, as well as osmolytes. Attachment capabilities by Δ*sagS*::CTX-*sagS* and Δ*sagS*::CTX were determined by crystal violet (CV) staining assay following 24 h of growth under shaking conditions at 37°C. C, negative control. The image was created using biorender.com. (B) From the initial Biolog screen, seven compounds increased attachment by Δ*sagS*::CTX-*sagS* relative to Δ*sagS*::CTX. The graph shows normalized attachment based on CV staining by the Δ*sagS*::CTX-*sagS* strain relative to Δ*sagS*::CTX mutant. Each compound was tested in biological duplicate with one well per assay. Error bars indicate standard deviations. (C and D) Compounds identified from the Biolog screen to enhance attachment by Δ*sagS*::CTX-*sagS* relative to Δ*sagS*::CTX were further evaluated for their ability to modulate attachment by Δ*sagS*::CTX and Δ*sagS*::CTX-*sagS* when grown in VBMM (C) and LB medium (D). Attachment was assessed after 24 h of growth under shaking conditions. Attachment is expressed relative to the strains grown in VBMM or LB alone. Experiments were carried out in triplicate using at least eight technical replicates each. Error bars indicate standard deviations. *, Statistically significant difference relative to untreated (–) (*P* < 0.01). Glc6P, glucose-6-phosphate; Gly, glycerol; β-MG, β-methyl-galactoside.

To identify potential ligands that may interact with and thus activate SagS, we made use of the Biolog phenotypic microarray assays. The assay was modified to screen for compounds that modulate attachment by P. aeruginosa in a SagS-dependent manner (Fig. 1A). To do so, we made use of Δ*sagS*::CTX-*sagS* (Δ*sagS* mutant complemented with a chromosomal *sagS* under the control of its own promoter), and the Δ*sagS* mutant harboring the empty pMini-CTX vector (Δ*sagS*::CTX) (34). These strains were chosen based on previous research demonstrating Δ*sagS*::CTX-*sagS* to be indistinguishable from the wild type, while Δ*sagS*::CTX was found to be comparable to a Δ*sagS* mutant strain (33, 34).

Attachment capabilities were analyzed after 24 h of incubation by crystal violet staining. We focused on compounds that modulated attachment by a *sagS* complemented strain (Δ*sagS*::CTX-*sagS*) but not a Δ*sagS*::CTX mutant strain. The screen revealed compounds that not only enhanced attachment by Δ*sagS*::CTX-*sagS* (Fig. 1B) but also reduced attachment (see Fig. S1 in the supplemental material) relative to Δ*sagS*::CTX. Reduced attachment was noted in the presence of a large number of compounds, with the most dramatic reduction in attachment noted upon exposure to β-hydroxy butyric acid, melbionic acid, oxalomalic acid, and *N*-acetyl-l-glutamic acid (see Fig. S1). In contrast, compounds enhancing attachment by Δ*sagS*::CTX-*sagS* but not Δ*sagS*::CTX included arabinose, l-proline, glycerol, d-glucose-6-phosphate, d-threonine, β-galacturonic acid, and β-methyl-d-galactoside (Fig. 1B).

10.1128/mSphere.01231-20.1 FIG S1 Compounds identified from the Biolog screen that coincided with reduced attachment by Δ*sagS*::CTX-*sagS* relative to Δ*sagS*::CTX mutant strain. Graphs show OD_570_/OD_600_ values of CV bound by the Δ*sagS*::CTX-*sagS* strain normalized to values obtained from the Δ*sagS*::CTX mutant. Each compound was tested in biological duplicate with one well per assay. Error bars indicate standard deviations. Download FIG S1, PDF file, 0.1 MB. Copyright © 2021 Park et al. 2021 Park et al. https://creativecommons.org/licenses/by/4.0/ This is an open-access article distributed under the terms of the Creative Commons Attribution 4.0 International license.

To ensure that the compounds identified in the Biolog indeed enhanced attachment in a SagS-dependent manner, we performed standard biofilm attachment assays in 96-well microtiter plates using two different media, Luria-Bertani (LB) medium and Vogel-Bonner minimal medium (VBMM), and the compounds at the concentration provided in the original Biolog assays. Under the conditions tested, we were unable to confirm whether l-proline, l-arabinose, d-threonine, and β-galacturonic acid have an effect on attachment (data not shown), and the respective compounds were eliminated from further studies. Glycerol was found to enhance attachment of Δ*sagS*::CTX when tested using VBMM medium (Fig. 1C) but enhanced attachment of both Δ*sagS*::CTX-*sagS* and Δ*sagS*::CTX when experiments were performed using LB medium (Fig. 1D). Since the finding suggested glycerol to stimulate attachment independent of SagS, the compound was likewise eliminated from further studies. β-Methyl-galactoside consistently enhanced attachment by Δ*sagS*::CTX but not Δ*sagS*::CTX-*sagS* regardless of the growth medium used (Fig. 1C and D). In contrast, exposure to glucose-6-phosphate had the opposite effect on attachment, with glucose-6-phosphate significantly enhancing the attachment by Δ*sagS*::CTX-*sagS* but not Δ*sagS*::CTX by up to 50% in VBMM and up to 30% in LB medium (Fig. 1C and D).

### Glucose-6-phosphate enhances attachment in a concentration-dependent manner.

**FIG 2:**
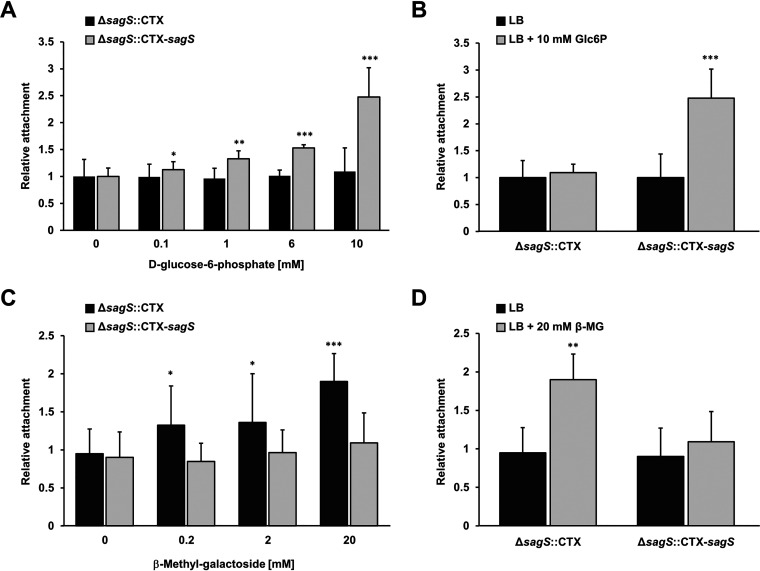
Effect of glucose-6-phosphate and β-methyl galactoside on SagS-mediated attachment. (A to D) Attachment by Δ*sagS*::CTX and Δ*sagS*::CTX-*sagS* in response to increasing concentrations of glucose-6-phosphate (Glc6P) (A and B) or β-methyl galactoside (β-MG) (C and D). Attachment assays were carried out in LB medium alone or supplemented with glucose-6-phosphate or β-methyl galactoside at the concentrations indicated, with attachment assessed using CV staining. Experiments were carried out in triplicate using at least eight technical replicates each. Error bars indicate standard deviations. Asterisks indicate statistically significant differences relative to treatment with LB medium alone (*, *P* < 0.05; **, *P* < 0.01; ***, *P* < 0.001).

Since glucose-6-phosphate at a concentration of 6.6 mM and β-methyl-galactoside at a concentration of 20.6 mM significantly modified attachment, with glucose-6-phosphate enhancing attachment only by Δ*sagS*::CTX-*sagS* and β-methyl-galactoside enhancing attachment by Δ*sagS*::CTX but not Δ*sagS*::CTX-*sagS*, we next determined whether the stimulatory effect by these two compounds is a concentration-dependent response. We therefore evaluated attachment by Δ*sagS*::CTX-*sagS* and Δ*sagS*::CTX in the presence of increasing concentrations of glucose-6-phosphate (0.1, 1, 6.6, and 10 mM) and β-methyl-galactoside (0.2, 2, and 20 mM). The absence or presence of glucose-6-phosphate, regardless of the concentration used, had no significant effect on attachment by Δ*sagS*::CTX (Fig. 2A). In contrast, glucose-6-phosphate significantly enhanced attachment by Δ*sagS*::CTX-*sagS* (Fig. 2A), with the stimulatory effect of glucose-6-phosphate being most apparent at 1, 6.6, and 10 mM (Fig. 2B). However, as little as 0.1 mM glucose-6-phosphate induced a significant difference in attachment (Fig. 2A). A similar stimulatory effect on attachment by glucose-6-phosphate was noted for wild-type P. aeruginosa (see Fig. S2A). In comparison, β-methyl-galactoside had the opposite effect, with increasing concentrations of β-methyl-galactoside enhancing the attachment of Δ*sagS*::CTX but not Δ*sagS*::CTX-*sagS* (Fig. 2C). Furthermore, exposure to β-methyl-galactoside had no effect on attachment by P. aeruginosa PAO1 (see Fig. S2B). The largest increase in attachment by Δ*sagS*::CTX was noted at 20 mM β-methyl-galactoside (Fig. 2D). Our findings suggest that although both glucose-6-phosphate and β-methyl-galactoside affect attachment, only glucose-6-phosphate enhances attachment in a manner dependent on SagS.

10.1128/mSphere.01231-20.2 FIG S2 Attachment by wild-type P. aeruginosa PAO1 in response to increasing concentrations of glucose-6-phosphate (A) or β-methyl galactoside (B). Attachment assays were carried out in LB medium alone or supplemented with glucose-6-phosphate or β-methyl-galactoside at the concentrations indicated, with attachment assessed using CV staining. Experiments were carried out in duplicate using at least eight technical replicates each. Error bars indicate standard deviations. Asterisks indicate statistically significant differences relative to LB medium alone (**, *P* < 0.05). Download FIG S2, PDF file, 0.1 MB. Copyright © 2021 Park et al. 2021 Park et al. https://creativecommons.org/licenses/by/4.0/ This is an open-access article distributed under the terms of the Creative Commons Attribution 4.0 International license.

### Glucose-6-phosphate does not enhance growth by *P. aeruginosa*.

To ensure that modulation of attachment was not related to the respective compounds increasing growth, we performed growth studies in the presence/absence of glucose-6-phosphate and β-methyl-galactoside. No difference in growth was noted for Δ*sagS*::CTX-*sagS* and Δ*sagS*::CTX when grown in LB or M9 minimal medium with glucose as a sole carbon source (see Fig. S3A). Likewise, neither glucose-6-phosphate nor β-methyl-galactoside had an effect on the growth (see Fig. S3B), indicating that enhanced biofilm formation is not due to enhanced growth. In addition, no growth was detected in M9 minimal medium when glucose as the only carbon source was substituted with glucose-6-phosphate or β-methyl-galactoside (see Fig. S3C), suggesting that these compounds do not serve as a carbon or energy source. Our findings of glucose-6-phosphate not supporting growth is in agreement with findings by Castañeda-García et al. (35) of P. aeruginosa lacking a specific glucose-6-phosphate transport system.

10.1128/mSphere.01231-20.3 FIG S3 (A) Growth curves of Δ*sagS*::CTX were compared to Δ*sagS*::CTX-*sagS* in either LB medium or M9 minimal medium supplemented with 0.2% glucose (Glc) as the sole carbon source. Growth behaviors by Δ*sagS*::CTX-*sagS* and Δ*sagS*::CTX when grown in LB medium in the absence or presence of 0.2% glucose-6-phosphate (Glc6P), β-methyl galactoside (β-MG), fructose-1,6-bisphosphate (Fur-1,6bp), glycerol (Gly), or proline (Pro) (B) and when grown in M9 minimal medium supplemented with the indicated compounds (C) as the sole carbon source. After inoculation, 200-μl aliquots of each strain were transferred into a 96-well plate, and growth was monitored by measuring the absorbance at 600 nm at 37°C in a multimode microplate reader (SpectraMax i3x plate reader; Molecular Devices). The means and standard deviations of four measurements are shown. Download FIG S3, PDF file, 0.2 MB. Copyright © 2021 Park et al. 2021 Park et al. https://creativecommons.org/licenses/by/4.0/ This is an open-access article distributed under the terms of the Creative Commons Attribution 4.0 International license.

### SagS-mediated biofilm formation is enhanced by glucose-6-phosphate.

**FIG 3:**
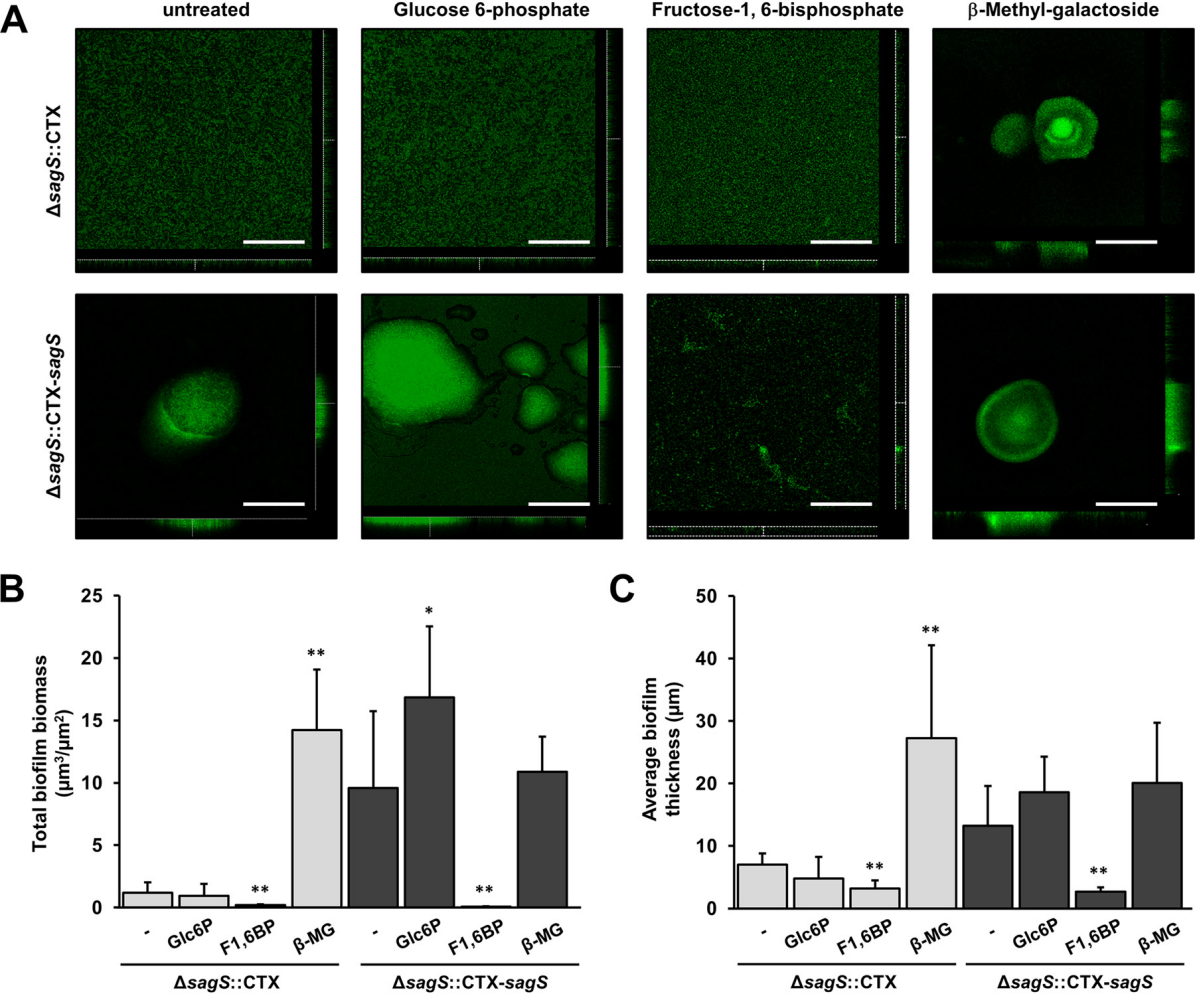
Glucose-6-phosphate enhances biofilm formation in a SagS-dependent manner. (A) Representative confocal microscopy images of the biofilm architecture by Δ*sagS*::CTX and Δ*sagS*::CTX-*sagS* strains. Biofilms were grown for 3 days in 5-fold-diluted LB medium supplemented with or without the indicated compounds and stained prior to microscopy using a Live/Dead *Bac*Light viability stain. Scale bars, 100 μm. The total biofilm biomass (B) and biofilm thickness (C) were determined by COMSTAT analysis. Experiments were carried out in triplicate with at least 10 images each. Error bars indicate standard deviations. Asterisks indicate statistically significant differences relative to biofilms for Δ*sagS*::CTX or Δ*sagS*::CTX-*sagS* strains tested in the absence of the indicated compounds (*, *P* < 0.05; **, *P* < 0.01). Glc6P, glucose-6-phosphate; F1,6BP, fructose-1,6-bisphosphate; β-MG, β-methyl-galactoside.

Our findings suggest that glucose-6-phosphate and β-methyl-galactoside might act as an input cue, with only glucose-6-phosphate likely mimicking conditions that are sensed by SagS and modulate or activate SagS activity. Since SagS has previously been demonstrated to not only affect attachment but also subsequent biofilm formation (24, 31, 32), we sought to determine whether glucose-6-phosphate likewise modulates biofilm formation in a SagS-dependent manner. To examine the effects of glucose-6-phosphate as a potential cue on biofilm formation, biofilms by Δ*sagS*::CTX-*sagS* and Δ*sagS*::CTX were allowed to grow in the absence or presence of glucose-6-phosphate for 3 days, with the resulting biofilm structure subsequently imaged by confocal scanning laser microscopy (CSLM). In agreement with previous findings (24, 34), biofilms formed by Δ*sagS* mutant cells appeared to be thin and unstructured while the complemented Δ*sagS*::CTX-*sagS* formed wild-type like, structured biofilms with large microcolonies approximately 100 μm in diameter (Fig. 3A; see also Fig. S4 in the supplemental material). The differences in biofilm architecture were confirmed by quantitative analysis of the biofilm biomass and biofilm height (Fig. 3B and C). Exposure of Δ*sagS*::CTX-*sagS* biofilms to glucose-6-phosphate coincided with an apparent increase in the number of microcolonies and microcolonies demonstrating increased diameters (Fig. 3A), significantly increased biofilm biomass (Fig. 3B) and biofilm height (Fig. 3C) relative to untreated Δ*sagS*::CTX-*sagS* biofilms. In contrast, glucose-6-phosphate had no effect on the Δ*sagS*::CTX biofilm architecture (Fig. 3).

10.1128/mSphere.01231-20.4 FIG S4 Representative confocal microscopy image of the biofilm architecture by P. aeruginosa PAO1. Biofilms were grown for 3 days in 5-fold-diluted LB medium and stained prior to microscopy using the Live/Dead *Bac*Light viability stain. Scale bars, 100 μm. Download FIG S4, PDF file, 0.1 MB. Copyright © 2021 Park et al. 2021 Park et al. https://creativecommons.org/licenses/by/4.0/ This is an open-access article distributed under the terms of the Creative Commons Attribution 4.0 International license.

We also evaluated the effect of β-methyl-galactoside on biofilm formation. Given that this compound enhanced the attachment of Δ*sagS*::CTX but not Δ*sagS*::CTX-*sagS* in a manner opposite to glucose-6-phosphate (Fig. 2), we anticipated β-methyl-galactoside to positively affect biofilm formation by *sagS*::CTX but not Δ*sagS*::CTX-*sagS.* As expected, exposure to β-methyl-galactoside enhanced biofilm architecture of Δ*sagS*::CTX without affecting Δ*sagS*::CTX-*sagS* biofilm formation (Fig. 3A). Our visual observations were confirmed by the quantitative analysis of the biofilm architecture (Fig. 3B and C), further confirming the stimulatory effect of β-methyl-galactoside being independent of SagS. In contrast, our findings indicated glucose-6-phosphate to enhance biofilm formation in a manner dependent on SagS, likely suggesting glucose-6-phosphate to be a specific cue of SagS.

### The hexose phosphate fructose-1,6-bisphosphate repressed biofilm formation by *P. aeruginosa* in a SagS-independent manner.

Glucose-6 phosphate is the pivotal intermediate of glucose metabolism and lies at the crossroads of different metabolic pathways, acting as a hub to metabolically connect glycolysis (Embden-Meyerhof, Entner-Doudoroff), the pentose phosphate pathway, glycogen synthesis, *de novo* lipogenesis, and the hexosamine pathway. Moreover, glucose-6-phosphate is very common in cells as glucose entering a cell is phosphorylated, with phosphorylation trapping glucose by placing a negative charge on the molecule, thus preventing its diffusion back across the cell membrane into the surrounding environment. To determine whether glucose-6-phosphate is a specific cue of SagS or whether its effect is related to its phosphorylation state, we sought to determine whether another hexose phosphate is likewise capable of enhancing biofilm formation in a SagS-dependent manner. We chose fructose-1,6-bisphosphate since this compound is a predominant glycolytic intermediate in species other than Gram-negative bacteria, including species of *Pseudomonas* that make use of the Entner-Doudoroff pathway. While exposure to fructose-1,6-bisphosphate had no apparent effect on the biofilm architecture of Δ*sagS*::CTX biofilms, exposure of Δ*sagS*::CTX-*sagS* to fructose-1,6-bisphosphate coincided with the formation of unstructured biofilms relative to untreated biofilms (Fig. 3A). Overall, Δ*sagS*::CTX-*sagS* biofilms exposed to fructose-1,6-bisphosphate were comparable to untreated Δ*sagS*::CTX biofilms (Fig. 3). Analysis of biofilms by CSLM combined with quantitative analysis of the biofilm structure demonstrated that fructose-1,6-bisphosphate significantly reduced biomass and average thickness by Δ*sagS*::CTX-*sagS*, as well as the *sagS*-deficient mutant strain, relative to untreated biofilms (Fig. 3B and C). These findings suggested that while fructose-1,6-bisphosphate negatively affects biofilm formation, its effect on biofilm biomass and biofilm thickness is opposite that of β-methyl-galactoside rather than glucose-6-phosphate, with fructose-1,6-bisphosphate likely not being perceived by SagS. The findings further indicate glucose-6-phosphate rather than the phosphorylation state to be perceived by SagS.

### Glucose-6-phosphate enhances biofilm marker gene expression in a SagS-dependent manner.

**FIG 4:**
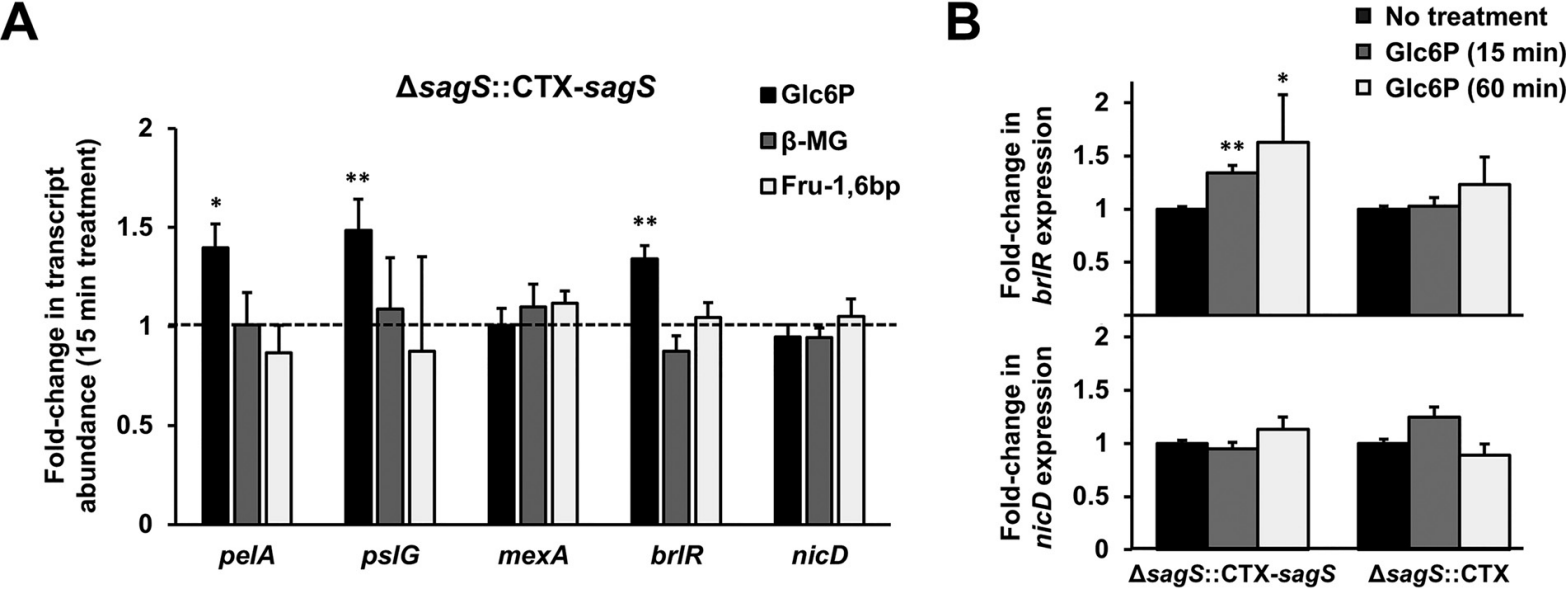
Effect of glucose-6-phosphate on the expression of the biofilm marker genes, as determined by qRT-PCR. Biofilms grown for 3 days in biofilm tube reactors were exposed to a 10 mM concentration of the indicated compound for 15 min (A) or 60 min (B) under flowing conditions. Postexposure, the biofilms were harvested immediately to isolate RNA. The relative expression levels of indicated biofilm marker genes were determined by qRT-PCR. The transcript abundance was normalized to the expression of Δ*sagS*::CTX-*sagS* in the absence of compound, with *mreB* being used as a housekeeping gene. Average data from three independent experiments are shown. Asterisks indicate statistically significant differences (*, *P* < 0.05; **, *P* < 0.01) from untreated biofilms. Glc6P, glucose-6-phosphate; β-MG, β-methyl-galactoside; Fru-1,6bp, fructose-1,6-bisphosphate.

The formation of biofilms has been reported to result with significant physiological changes, with the transition from the planktonic to the sessile lifestyle coinciding with changes at the transcriptional level (7). In agreement with these findings, we previously demonstrated that SagS enhances the transcript abundance of several biofilm marker genes, including *pelA* and *pslG*, encoding Pel and Psl glycoside hydrolases, respectively, but represses the expression of genes linked to motility (*fliC*, *pilY1*) (34). Moreover, Δ*sagS* mutant biofilms were characterized by reduced *brlR* transcript, and the transcriptional regulator of biofilm-specific antibiotic tolerance BrlR activates the expression of *mexA* encoding a component of the multidrug efflux pump (26, 36). Given that glucose-6-phosphate enhanced attachment and biofilm formation in a SagS-dependent manner, with glucose-6-phosphate likely contributing to the activation of SagS, we reasoned that sensing of glucose-6-phosphate by SagS would have a positive effect on the transcript abundance of biofilm marker genes. To address our hypothesis, biofilms formed by Δ*sagS*::CTX-*sagS* were exposed to 10 mM glucose-6-phosphate for 15 min or left untreated, and the expression levels of *pelA* and *pslG* were subsequently determined by qRT-PCR. As expected, a positive correlation between the degree of biofilm marker genes expression and biofilm formation was noted. Under the conditions tested, *pelA* and *pslG* were upregulated upon exposure to glucose-6-phosphate (Fig. 4A). Likewise, increased transcript abundance of *brlR* but not *mexA* was noted in the presence of glucose-6-phosphate (Fig. 4A). It is of interest to note that extended exposure to glucose-6-phosphate for 1 h coincided with a further increase in *brlR* transcript abundance by Δ*sagS*::CTX-*sagS* biofilms, a response that was absent in biofilms formed by Δ*sagS*::CTX (Fig. 4B).

To ensure that the noted increase in the expression was specific to glucose-6-phosphate, we also evaluated transcript abundance postexposure to fructose-1,6-bisphosphate and β-methyl-galactoside. No significant difference in *pelA*, *pslG*, *brlR*, and *mexA* expression was noted in the presence of fructose-1,6-bisphosphate or β-methyl-galactoside (Fig. 4A), further confirming SagS to specifically respond to and be activated by glucose-6-phosphate.

### Glucose-6-phosphate enhances c-di-GMP levels in biofilms in a SagS-dependent manner.

**FIG 5:**
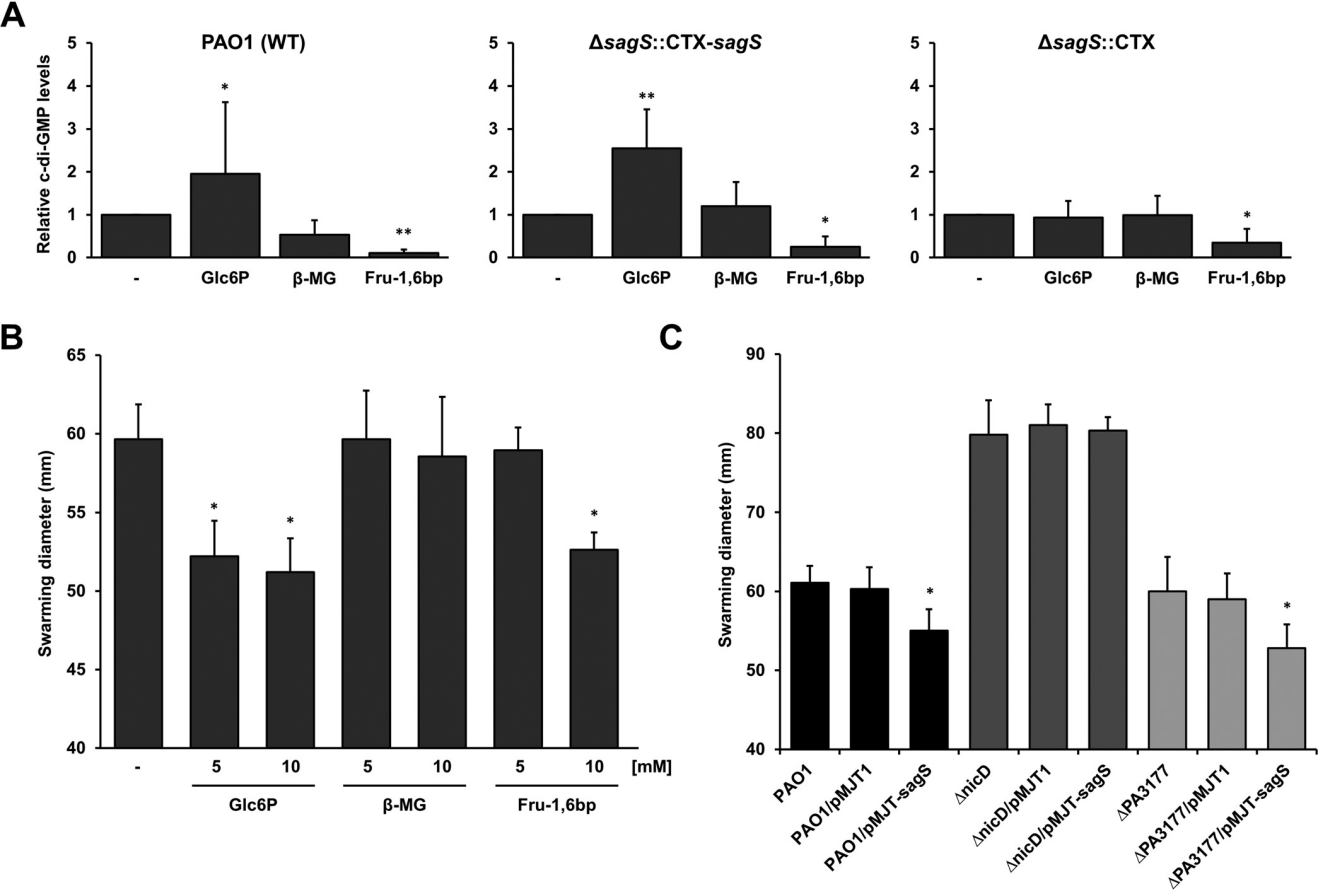
Glucose-6-phosphate stimulates increases in intracellular c-di-GMP in a SagS-dependent manner. (A) Relative levels of intracellular c-di-GMP present biofilms formed by wild-type P. aeruginosa PAO1, Δ*sagS*::CTX-*sagS*, and Δ*sagS*::CTX cells harboring the unstable c-di-GMP reporter pCdrA::*gfp*(ASV) and the pMF440-based mCherry expression plasmid. Biofilms were grown for 4 days in 5-fold-diluted LB medium supplemented with glucose-6-phosphage (Glc6P), β-methyl-galactoside (β-MG), or fructose-1,6-bisphosphate (Fru-1,6bp). The fluorescence of each biofilms was then measured. Relative green fluorescence units are arbitrary fluorescence intensity units corrected for red fluorescence units. Asterisks indicate statistically significant differences (*, *P* < 0.05; **, *P* < 0.01) from untreated biofilms. (B) Swarming motility of wild-type P. aeruginosa PAO1 cells in the absence or presence of glucose-6-phosphage (Glc6P), β-methyl-galactoside (β-MG), or fructose-1, 6-bisphosphate (Fru-1,6bp) at the indicated concentrations. Ansterisk (*) indicates a statistically significant difference (*P* < 0.01) from untreated cells. (C) Swarming motility of wild-type P. aeruginosa PAO1, Δ*nicD* mutant, and ΔPA3177 mutant cells harboring the empty vector pMJT-1 or expressing *sagS*. *, Significantly different relative to the respective strain harboring the empty vector (*P* < 0.05). Experiments were carried out in triplicate using at least 10 images each. Error bars indicate standard deviations.

The second messenger c-di-GMP is one of the key modulators of the transition to biofilms in diverse bacterial species (20). While low intracellular levels of c-di-GMP promote a motile lifestyle, high levels have been associated with biofilm formations (37). We previously demonstrated that inactivation of *sagS* coincided with reduced cellular levels of c-di-GMP relative to wild-type biofilms, with complementation restoring c-di-GMP to wild-type levels (29, 31). Since biofilm c-di-GMP levels are linked to SagS (32, 38) and exposure to glucose-6-phosphate coincided with the formation of more substantial biofilms in a SagS-dependent manner (Fig. 3), we next sought to determine whether the altered biofilm architecture is due to elevated levels of c-di-GMP. We therefore made use of an unstable green fluorescent protein (GFP) reporter [PcdrA::*gfp*(ASV)] for which the fluorescence intensity is directly proportional to the concentration of intracellular c-di-GMP (39) to determine levels of c-di-GMP in the absence or presence of glucose-6-phosphate. Biofilms formed by wild-type and Δ*sagS*::CTX-*sagS* displayed on average a 2.5-fold increase in the level of c-di-GMP in the presence of glucose-6-phosphate compared to untreated biofilms (Fig. 5A), while no differences in c-di-GMP levels were noted for biofilms formed by the *sagS*-deficient mutant (Δ*sagS*::CTX) in the absence or presence of glucose-6-phosphate (Fig. 5A). In contrast, exposure to fructose-1,6-bisphosphate coincided with significantly reduced intracellular level of c-di-GMP by all strains tested (Fig. 5A). The findings are in agreement with the significant difference in the biofilm architecture formed in the presence of either of the two compounds (Fig. 3). Interestingly, while the addition of β-methyl-galactoside enhanced biofilm formation (Fig. 3B and C) by Δ*sagS*::CTX, no significant differences in c-di-GMP level were noted in the absence or presence of β-methyl-galactoside (Fig. 5A), suggesting that β-methyl-galactoside-mediated biofilm formation is not driven by increased c-di-GMP levels.

Swarming motility, a surface-associated motile behavior, has been used as an indirect indicator for c-di-GMP levels, with high intracellular levels of c-di-GMP reducing the swarming motility (20, 37). We therefore hypothesized that if glucose-6-phosphate indeed induces an increase in cellular c-di-GMP levels, then the presence of this compound will coincide with a decrease in swarming motility. We therefore inoculated approximately 10^6^ wild-type cells on swarming medium supplemented with increasing concentrations of glucose-6-phosphate. Swarming medium lacking glucose-6-phosphate was used as negative control. Increasing concentrations of glucose-6-phosphate coincided with decreasing swarming motility in wild-type P. aeruginosa (Fig. 5B). These data suggest a link between glucose-6-phosphate and the modulation of the cellular levels of c-di-GMP.

### SagS exerts its function in modulating c-di-GMP via NicD.

Our findings so far suggest that exposure of glucose-6-phosphate coincides with significantly increased c-di-GMP levels and induction of biofilm-related phenotypes, with the response being dependent on SagS. SagS has been previously linked to the elevated levels of c-di-GMP present in P. aeruginosa biofilm (29). This is supported by the finding that biofilms formed by Δ*sagS* mutants were characterized by low c-di-GMP levels relative to wild-type biofilms that were comparable to those found in planktonic cells (26). Given our finding of SagS-dependent modulation of c-di-GMP present in biofilms in response to glucose-6-phosphate (Fig. 5A) despite SagS lacking c-di-GMP modulating domains such as GGDEF, and EAL or HD-GYP that are involved in c-di-GMP synthesis and hydrolysis, respectively (31), we reasoned that SagS indirectly regulates c-di-GMP levels via a distinct c-di-GMP modulating protein. To determine which c-di-GMP-modulating protein is stimulated by SagS, we focused on two c-di-GMP-modulating enzymes, NicD and PA3177. NicD has been identified as a nutrient-responsive diguanylate cyclase (DGC) that is capable of sensing dispersion-inducing nutrient cues, including glutamate and succinate (40), while PA3177 has been characterized as an active DGC, which is required to activate the c-di-GMP responsive transcriptional regulator BrlR and thus biofilm drug tolerance (38). Both DGCs have been demonstrated to contribute the cellular c-di-GMP level present in biofilms (38, 40). We reasoned that if one or both DGCs work in concert with SagS to affect c-di-GMP levels in a glucose-6-phosphate-dependent manner, expression of *sagS* in a Δ*nicD* or ΔPA3177 mutant background would not result in decreased swarming in the presence of glucose-6-phosphate, as noted upon overexpression of *sagS* in wild-type P. aeruginosa PAO1. However, if the DGCs contribute to c-di-GMP levels independently, decreased swarming was anticipated. As anticipated, a PAO1 strain overexpressing *sagS* (PAO1/pMJT-*sagS*) exhibited decreased swarming motility compared to the wild-type strain carrying an empty vector in the presence of glucose-6-phosphate (Fig. 5C). Similar results were obtained upon overexpression of *sagS* in an isogenic PA3177 mutant strain (Fig. 5C). In contrast, no difference in swarming motility was noted in a *nicD*-deficient mutant strain harboring an empty vector or overproducing SagS. The findings indicated that SagS-dependent swarming motility in the presence of glucose-6-phosphate is dependent on the DGC activity by NicD.

### The stimulatory effect of glucose-6-phosphate on biofilm formation is independent of NicD.

**FIG 6:**
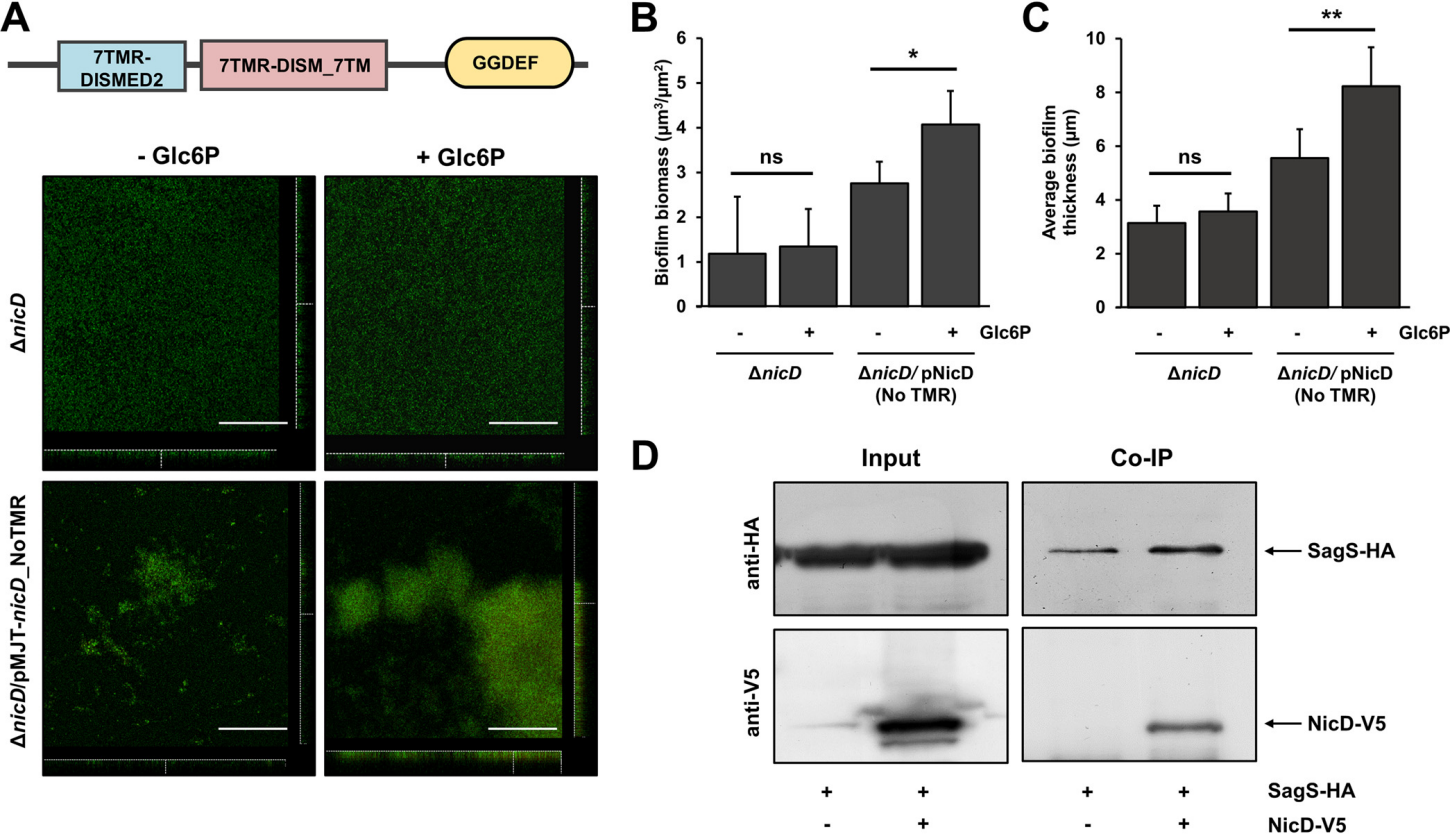
SagS modulates the intracellular c-di-GMP pool via interaction with NicD. (A, top panel) Schematic presentation of the NicD polypeptide. NicD is an active diguanylate cyclase that harbors three domains, including a periplasmic 7TMR-DISMED2 sensory domain, a membrane-spanning 7TMR-DISM_7TM domain, and a cytoplasmic GGDEF domain. (Bottom panel) Representative confocal images showing the architecture of biofilms formed by Δ*nicD* and Δ*nicD* mutant strains overexpressing a truncated NicD construct lacking the N-terminal sensory TMR domain (NicDΔ_NoTMR) under the control of the arabinose-inducible promoter of the pMJT1 vector. Biofilms were grown for 3 days in 5-fold-diluted LB medium supplemented with or without glucose-6-phosphate (Glc6P) and stained prior to microscopy using Live/Dead *Bac*Light viability stain. Scale bars, 100 μm. The total biofilm biomass (B) and biofilm thickness (C) were determined by COMSTAT analysis. Experiments were carried out in triplicate using at least 10 images each. Error bars indicate standard deviations. Asterisks indicate statistically significantly differences (*, *P* < 0.05; **, *P* < 0.01) from untreated biofilms. ns, not significant. (D) Pulldown assay using HA-tagged SagS as bait. Copurification of V5-tagged NicD was detected by immunoblotting with anti-V5 antibodies. The data are representative of three independent experiments, all of which gave similar results.

NicD is composed of an extracellular 7TMR-DISMED2 (7 Trans-Membrane Receptor with Diverse Intracellular Signaling Modules extracellular) domain, a membrane-spanning 7TMR DISM_7TM (7TMR-DISM, 7 Trans-Membranes) domain, followed by a GGDEF domain typically associated with cytoplasmic diguanylate activity (Fig. 6A) (40). As the periplasmic 7TMR-DISMED2 domain of NicD has been linked to cue sensing by directly binding carbohydrates, with binding having been reported to coincide with increased diguanylate cyclase activity (40), it is likely that NicD rather than SagS directly perceives glucose-6-phosphate and thus contributes to the modulation of c-di-GMP levels by directly responding to glucose-6-phosphate. We reasoned that if NicD rather than SagS is involved in sensing glucose-6-phosphate, removal of the periplasmic sensory domain 7TMR-DISMED2 would abolish the glucose-6-phosphate-induced enhancement of biofilm formation. It is interesting to note that the truncated NicD variant retains its diguanylate cyclase activity (40).

We therefore evaluated the biofilm architecture by Δ*nicD* and a Δ*nicD* mutant strain producing a NicD variant lacking the periplasmic 7TMR-DISMED2 domain (Δ*nicD*/*nicD_*NoTMR) grown in the presence or absence of glucose-6-phosphate. In agreement with previous findings (40), biofilms formed by the Δ*nicD* mutant appeared to be thin and unstructured (Fig. 6A), with glucose-6-phosphate having no effect on the overall biomass accumulation of thickness of the mutant biofilm (Fig. 6B and C). In contrast, a Δ*nicD* mutant producing a truncated NicD variant lacking the periplasmic 7TMR-DISMED2 domain, formed wild-type like, structured biofilms (Fig. 6A). The presence of glucose-6-phosphate coincided with an apparent increase in the biofilm structure by Δ*nicD*/*nicD_*NoTMR (Fig. 6A), which was supported by the quantitative analysis of the biofilm biomass (Fig. 6B) and the average biofilm thickness (Fig. 6C) relative to the absence of glucose-6-phosphate. The findings indicated that while NicD contributed to the overall increase in biofilm formation in response to glucose-6-phosphate, the response was independent of the periplasmic sensory domain of NicD.

### SagS interacts with NicD.

Considering that NicD does not appear to be directly involved in glucose-6-phosphate sensing but its activity significantly contributes to the increase in biofilm biomass accumulation noted in the presence of glucose-6-phosphate, we next sought to determine how SagS accomplishes the modulation of the intracellular c-di-GMP pool via NicD. Since exposure to glucose-6-phosphate only coincided with minimal change in *nicD* transcript abundance (Fig. 4), we investigated whether SagS affects NicD posttranscriptionally. We therefore determined whether SagS physically interacts with NicD. To do so, we performed a coimmunoprecipitation assay of full-length proteins using P. aeruginosa PAO1 cells producing a C-terminally hemagglutinin (HA)-tagged SagS (SagS_HA) and C-terminally V5-tagged NicD (NicD_V5). Antibodies recognizing the HA epitope pulled down NicD_V5 (Fig. 6D), indicating that SagS interacts with the DGC NicD.

## DISCUSSION

Bacterial biofilm formation is a regulated process that is driven by the coordinated work of regulatory proteins within large signaling networks. P. aeruginosa can perceive and integrate various signals into signaling relays that modulate the transitions between motile and sessile modes of growth. One of the regulatory proteins, the hybrid sensor kinase SagS, promotes the switch from planktonic to biofilm growth mode by stimulating attachment and biofilm formation via hierarchical phosphotransfer to BfiS (24) and the switch from a susceptible to a tolerant state by indirectly activating the transcriptional regulator, BrlR (26, 31). Here, we explored small molecules responsible for the activation of SagS. Our screen revealed several compounds capable of modulating the switch between the motile and sessile lifestyle, including β-methyl-galactoside and glucose-6-phosphate. However, our findings indicate that β-methyl-galactoside serves as a cue in a manner independent of SagS. It is likely that the effect of β-methyl-galactoside on biofilm formation is linked to LecA. The galactophilic lectin LecA has been demonstrated to contribute to biofilm formation *in vitro* (41) and adhesion to lung tissues and, consequently, biofilm formation and P. aeruginosa-mediated alveolar destruction in an *in vivo* mice model of infection (42). Derivates of galactose, including methylated derivate such as β-methyl-galactoside, which mimic terminal sugars of eukaryotic cell surface glycoconjugates have been identified as lectin-inhibiting carbohydrates (42). However, lectin-inhibiting carbohydrates have also been reported to result in lectin-mediated aggregation of bacteria and subsequent formation of microcolonies (43). Our findings of β-methyl-galactoside restoring attachment and biofilm formation by P. aeruginosa strains inactivated in *sagS* are in agreement with lectin-inhibiting carbohydrates enhancing microcolony formation (Fig. 3).

In contrast to β-methyl-galactoside, glucose-6-phosphate was found to enhance attachment and biofilm formation by P. aeruginosa in a manner dependent on SagS (Fig. 1 and 3). Moreover, exposure to glucose-6-phosphate resulted in decreased swarming motility but increased intracellular c-di-GMP levels in biofilms (Fig. 5). This SagS-mediated regulation of c-di-GMP levels in response to glucose-6-phosphate was found to be governed by the DGC NicD. Since NicD is not directly involved in glucose-6-phosphate sensing (Fig. 6), it is possible that NicD contributes to c-di-GMP modulation in response to or via interaction with SagS; however, direct evidence in support of the interaction affecting NicD activity is lacking. Regardless, our results suggest that glucose-6-phosphate functions as a cue to activate SagS or mimics conditions sensed by SagS to enhance the mode of biofilm growth and c-di-GMP modulation. Overall, our findings presented here suggest that glucose-6-phosphate is an external cue capable of activating SagS to modulate attachment and biofilm formation, with SagS forming a complex with the diguanylate cyclase NicD to regulate cellular c-di-GMP level in biofilms as part of a signaling cascade in response to glucose-6-phosphate.

Glucose-6-phosphate is one of many host-derived hexose phosphates that have been identified as signals by pathogenic bacteria to recognize the host environment and, as carbon sources that enable intracellular growth, to subsequently modulate cellular processes, including survival and biofilm formation upon infection. For instance, the relationship between host-driven hexose phosphates, sensing, and bacterial survival in the host organ have been elucidated in the human-pathogenic bacterium Listeria monocytogenes (44). The intracellular pathogen recognizes and takes up glucose-6-phosphate via the Hpt permease, a bacterial homolog of the mammalian translocase, and utilizes it as a carbon source for adaptation and propagation within the host cytosol and ensuring survival within mouse organs. Similarly, the intracellular pathogen Francisella tularensis that is capable of replicating to high densities within the cytoplasm of infected cells in more than 250 known host species, including humans, relies on host-derived phosphates for survival (45). This is supported by the findings by Radlinski et al. (45) demonstrating that while F. tularensis is adept at modulating its metabolism to fluctuating concentrations of host-derived nutrients, the enzymes fructose 1,6-bisphosphatase (GlpX) and glycerol 3-phosphate dehydrogenase (GlpA) are essential for F. tularensis intracellular replication regardless of the infection models tested. While P. aeruginosa does not exploit hexose phosphates for growth (see Fig. S3), it is likely that SagS sensing of glucose-6-phosphate is an adaptation by P. aeruginosa to the host environment to initiate or enhance the formation of biofilms (Fig. 1 and 3). This is supported by the finding that P. aeruginosa has been shown to respond to host-derived compounds. Riquelme et al. (46) demonstrated that utilization of the host-derived compound itaconate redirected the metabolism by P. aeruginosa to promote biofilm formation, while Rao et al. (47) demonstrated host-derived inflammatory phospholipids to modulate the expression of *rahU* (PA0122), a novel oxidized low-density lipoprotein (LDL), and lysophosphatidylcholine binding protein in P. aeruginosa (48), which in turn reduced biofilm formation. Although we did not evaluate the contribution of glucose-6-phosphate on the pathogenicity by P. aeruginosa *in vivo*, SagS has been previously reported to contribute to the pathogenicity and the acute-to-chronic virulence switch using a murine model of chronic pneumonia (34). In addition, several lines of evidence suggest glucose-6-phosphate to be present in the niches where P. aeruginosa is capable of growing. For one, inflammatory reactions present in the lung environment, as well as wound sites, have been reported to coincide with cell damage and the release of intracellular contents (49, 50). Moreover, glucose-6-phosphate dehydrogenase (G6PD) deficiency is a genetic metabolic abnormality caused by deficiency of the enzyme G6PD and the most common human enzymopathy, affecting upward of an estimated 400 million people worldwide (51). G6PD deficiency has been linked to increased susceptibility to infection and absent NETosis (52, 53).

Responses to external cues and host-derived compounds have been linked to the modulation of c-di-GMP, with several studies linking the interplay to TCSs. During infection of the host plant *Brassica oleracea* cv. Zhonggan 11 by Xanthomonas campestris, c-di-GMP interacts with the histidine kinase RavS, the interaction coinciding with decreased phosphorylation levels of RavS to hinder swimming motility. Outside the host, however, the X. campestris cognate response regulator of RavS, RavR, which harbors an active EAL domain, is activated to degrade c-di-GMP and thus enhances swimming motility (54). A similar interplay between TCS components of the Gac/Rsm pathway and c-di-GMP modulation has been described for P. aeruginosa PAK. Mutation of the component of Gac/Rsm cascade, RetS, exhibits a hyperbiofilm phenotype and elevated levels of c-di-GMP, while deletion of *sadC* encoding the DGC SadC in a *retS* mutant background leads to restoration to wild-type levels (55). Our finding of SagS enhancing c-di-GMP levels in response to glucose-6-phosphate is in agreement with previously reported interplays between TCS and c-di-GMP. Previous reports indicated that wild-type P. aeruginosa PAO1 and Δ*bfiS* biofilm cells contained on average 75 to 78 pmol/mg c-di-GMP, whereas c-di-GMP levels in Δ*sagS* biofilm cells were decreased to 33 ± 2 pmol/mg, suggesting that SagS is associated with the elevated levels of c-di-GMP present in biofilm (29). Since SagS does not contain enzymatic output domains, it can be assumed that a distinct c-di-GMP modulating protein might be related to the biofilm formation governed by the SagS-mediated signaling pathway. Here, we demonstrate that c-di-GMP modulation and the interplay between SagS and c-di-GMP in response to glucose-6-phosphate relies on the DGC NicD. Evidence supporting a role of NicD in this interplay stems from the finding that SagS and NicD interact to modulate biofilm formation, swarming motility, and c-di-GMP (Fig. 5 and 6). Thus, our findings provide another example of a cross talk between two-component regulatory systems and c-di-GMP signaling.

Although we showed several lines of evidence indicating that SagS-mediated biofilm formation and regulation of c-di-GMP levels are activated by glucose-6-phosphate, it still remains to be determined whether glucose-6-phosphate directly interacts with the periplasmic sensory domain HmsP of SagS. The interaction between SagS and glucose-6-phosphate will be the subject of future investigations. Likewise, given the effect of glucose-6-phosphate on *brlR* expression (Fig. 4), future studies will explore the contribution of glucose-6-phosphate on BrlR abundance and thereby the antibiotic tolerance of P. aeruginosa biofilms. Therefore, more experiments are needed to investigate the specific mechanism of how SagS senses glucose-6-phosphate and thus contributes to its dual functions.

Similar to other pathogens, however, that sense and respond to signals and cues present in their environment, including host-derived small molecules to modulate the expression of their virulence repertoire, our findings clearly demonstrate that the opportunistic pathogen P. aeruginosa senses and responds to glucose-6-phosphate to modulate biofilm formation and, by extension, other virulence related factors. That glucose-6-phosphate is likely made available following cell lysis raises the possibility that sensing and responding to glucose-6-phosphate is an adaptation to the host environment, likely after the induction of tissue damage, to stimulate biofilm formation and persistence in the host.

## MATERIALS AND METHODS

### Bacterial strains, plasmids, and culture conditions.

Bacterial strains, plasmids, and oligonucleotides used in this study are listed in Tables S1 and S2 in the supplemental material. Planktonic cells were grown at 37°C in LB medium or M9 minimal medium (pH 7.2) supplemented with the indicated carbon sources. Biofilms were grown as indicated below. The following supplements were added in all planktonic culture if necessary: tetracycline, 60 μg/ml for P. aeruginosa and 20 μg/ml for E. coli; gentamicin, 75 μg/ml for P. aeruginosa and 20 μg/ml for E. coli; carbenicillin, 250 μg/ml for P. aeruginosa; ampicillin, 100 μg/ml for E. coli; kanamycin, 50 μg/ml for E. coli; and l-arabinose, 0.1%.

10.1128/mSphere.01231-20.5 TABLE S1 Strains and plasmids used in this study. Download Table S1, PDF file, 0.2 MB. Copyright © 2021 Park et al. 2021 Park et al. https://creativecommons.org/licenses/by/4.0/ This is an open-access article distributed under the terms of the Creative Commons Attribution 4.0 International license.

10.1128/mSphere.01231-20.6 TABLE S2 Oligonucleotides used in this study. Download Table S2, PDF file, 0.09 MB. Copyright © 2021 Park et al. 2021 Park et al. https://creativecommons.org/licenses/by/4.0/ This is an open-access article distributed under the terms of the Creative Commons Attribution 4.0 International license.

### Screening of compounds using the Biolog system.

To identify potential molecules that would affect attachment in a SagS-dependent way, a high-throughput screening was performed using phenotyping microarray (PM) plates (Biolog, Inc., Hayward, CA) containing various carbon and nitrogen sources as well as osmolytes. For plates PM1 to PM3 (containing carbon and nitrogen sources), overnight cultures were diluted 100-fold in inoculation fluid IF-0 (Biolog) supplemented with 20 mM sodium succinate. Each well of the PM plate was filled with 100 μl of the diluted culture, followed by incubation at 37°C for 24 h with intermittent shaking at 220 rpm. For plate PM9 (osmolytes), overnight cultures were first diluted 100-fold in inoculation fluid IF-0 and subsequently 200-fold in inoculation fluid IF-10 without the addition of sodium succinate. Similarly, the plates were incubated at 37°C for 24 h with shaking. On day 2, the optical density at 600 nm (OD_600_) was first determined, followed by removal of the culture medium and, hence, nonattached cells. Each well was filled with 100 μl of 0.85% saline, to which 25 μl of a 0.1% (wt/vol) crystal violet solution was added. Plates were incubated for 15 min with shaking at 37°C, washed four times with 100 μl of Nanopure water, and air dried; the remaining crystal violet was then dissolved in 100 μl of 95% ethanol. Finally, the OD_570_ was determined using a SpectraMax i3x plate reader (Molecular Devices).

### Attachment assays.

Overnight cultures of P. aeruginosa were diluted 100-fold in LB medium supplemented with or without compounds of interest to an OD_600_ of 0.025, and 200 μl of the resulting dilution was added to each well of a 96-well plate, followed by 24 h of incubation at 37°C with continuous shaking at 220 rpm. Next, 50 μl of a 0.1% (wt/vol) crystal violet solution was added to each well, and the plates were incubated for 15 min at 37°C with shaking. Plates were washed four times with 200 μl of Nanopure water to remove nonattached cells and excess crystal violet and then allowed to dry. Finally, the remaining crystal violet was resuspended in 200 μl of 95% ethanol, and the OD_570_ was determined.

### Biofilm growth.

P. aeruginosa biofilms were grown in 20-fold-diluted LB medium using a continuous flow tube reactor system (1-m long, size 13 silicone tubing; Masterflex; Cole Parmer, Inc.) with an inner surface area of 25 cm^2^ at a flow rate of 0.1 ml/min (7, 56) in flow cell reactors (BioSurface Technologies). For the plate biofilm cultivation, biofilms were grown in 24-well plates in 5-fold-diluted LB medium supplemented with compounds of interest, as previously described (32, 33). The plates were incubated at 37°C and 220 rpm at a 30° angle, ensuring that the bottom of the wells is at the air-liquid interface and the location of biofilm formation. The medium was exchanged every 12 h. To maintain plasmids, carbenicillin at 10 μg/ml or gentamicin at 2 μg/ml was added to the growth medium. CLSM images were acquired using a Leica TCS SP5 confocal microscope (Leica Microsystems, Inc.) and the Live/Dead BacLight bacterial viability kit (Life Technologies, Inc.). Quantitative analysis of the confocal laser scanning microscope images of 24-well plate-grown biofilms was performed using COMSTAT (32, 33).

### RNA isolation and qRT-PCR.

Biofilms grown in biofilm tube reactors were harvested by extrusion, with the cell paste being collected directly into 500 μl of RNAprotect bacterial reagent (Qiagen), and total RNA was isolated using a E.Z.N.A. Total RNA kit (Omega Bio-Tek) according to the manufacturer’s instructions. Genomic DNA was removed using Turbo DNase (Thermo Fisher Scientific) for 30 min. The same amount of RNA (1,000 ng) from each cell was converted to cDNA using the iScript Select cDNA synthesis kit (Bio-Rad). qRT-PCR was performed using the Bio-Rad CFX Connect real-time PCR detection system and SsoAdvanced SYBR green Supermix (Bio-Rad) with the specific primers listed in Table S2. To normalize the transcript level, the *mreB* gene was used as a reference.

### Quantification of c-di-GMP.

For measuring the relative c-di-GMP levels, we used a dual fluorescence-based assay by using cells introducing plasmids expressing GFP(ASV) from the c-di-GMP responsive *cdrA* promoter [pCdrA::gfp(ASV)] (39) and expressing mCherry from a constitutive promoter. After biofilms were grown 4 days in 24-well plates as described above, the plates were washed with 0.85% saline to remove planktonic cells. Biofilms were then harvested by scraping into 0.85% saline and each fluorescence (GFP, 485 nm/535 nm; mCherry, 580 nm/620 nm) was measured in a 96-well black clear-bottom microtiter plate (Greiner Bio-One) using a SpectraMax i3x plate reader (Molecular Devices). Quantifications were performed in triplicate, and the fluorescence unit from GFP was normalized to mCherry.

### Swarming motility.

Swarming motility was determined using M8 minimal medium (Na_2_HPO_4_ [6 g/liter], KH_2_PO_4_ [3 g/liter], and NaCl [0.5 g/liter]) supplemented with 0.5% Casamino Acids, 0.2% glucose, 1 mM MgSO_4_, and 0.5% agar as described previously (38). After incubation at 37°C, swarming was determined by measuring the diameter of growth from the point of inoculation.

### Western blot analysis.

Crude extracts were prepared and the protein concentrations were determined using a modified Lowry assay with bovine serum albumin as a standard, as previously described (31). Samples were separated on a 10% SDS-polyacrylamide gel and subsequently transferred onto polyvinylidene difluoride membranes using a TurboTransblot apparatus (Bio-Rad). Then samples were analyzed by Western blotting using antibodies recognizing HA (BioLegend; 1:5,000) or V5 (Invitrogen; 1:5,000). Secondary horseradish peroxidase-conjugated antisera recognizing rabbit or mouse antibodies (Cell Signaling Technology) were used at 1:5,000 dilutions. The blots were developed using ImmunStar WesternC chemiluminescent reagents (Bio-Rad). After transfer, SDS-PAGE gels were Coomassie blue stained to ensure equal loading.

### Immunoprecipitation assay.

The interaction between SagS and NicD was investigated in wild-type P. aeruginosa PAO1 coproducing C-terminally HA and V5/His_6_-tagged SagS (full-length) and NicD (full-length), respectively. Cells grown in LB medium supplemented with 0.2% l-arabinose were harvested and resuspended in lysis buffer (50 mM Tris-HCl [pH 7.5], 150 mM NaCl, 1 mM EDTA, 0.2 mM phenylmethylsulfonyl fluoride) and disrupted by sonication. For a pulldown assay, crude extracts were incubated with antibodies recognizing either HA or V5 and protein A/G PLUS-Agarose (Santa Cruz Biotechnology) at 4°C for 2 h according to the manufacturer’s instructions. After washing the beads, the bound proteins were eluted in SDS loading buffer, separated on a 10% SDS-PAGE gel, and analyzed by Western blotting using anti-HA and anti-V5 antibodies.

### Statistical analysis.

All statistical analyses were performed in Microsoft Excel by using a two-tailed Student *t* test assuming equal variance or using single-factor analysis of variance. Unless otherwise noted, all experiments were performed at least in triplicate using biological replicates.
