# Supplementary material for: Glucose-6-Phosphate Acts as an Extracellular Signal of SagS To Modulate Pseudomonas aeruginosa c-di-GMP Levels, Attachment, and Biofilm Formation
Source: mSphere. 2021 Feb 10;6(1):e01231-20. doi: 10.1128/mSphere.01231-20 (PMC8544897; doi:10.1128/mSphere.01231-20)
Supplement: FIG S1 [file msphere.01231-20-sf001.pdf]

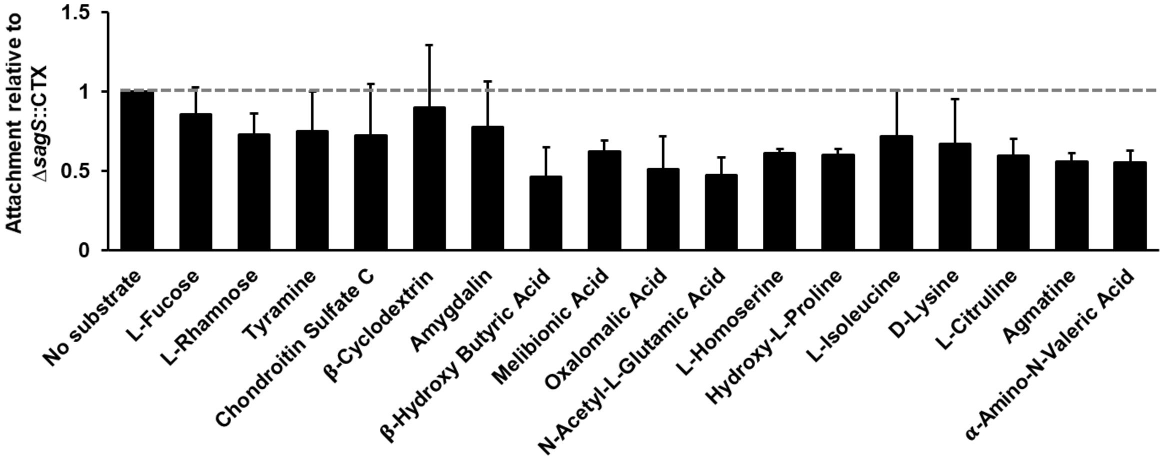

Figure S1. Compounds identified from the Biolog screen that coincided with reduced attachment by Δ*sagS*::CTX-*sagS* relative to Δ*sagS*::CTX mutant strain. Graph show OD_{570nm}/OD_{600nm} values of CV bound by the Δ*sagS*::CTX-*sagS* strain normalized to values obtained from the Δ*sagS*::CTX mutant. Each compound was tested in biological duplicate with one well per assay. Error bars indicate standard deviation.
