# Supplementary material for: Glucose-6-Phosphate Acts as an Extracellular Signal of SagS To Modulate Pseudomonas aeruginosa c-di-GMP Levels, Attachment, and Biofilm Formation
Source: mSphere. 2021 Feb 10;6(1):e01231-20. doi: 10.1128/mSphere.01231-20 (PMC8544897; doi:10.1128/mSphere.01231-20)
Supplement: FIG S2 [file msphere.01231-20-sf002.pdf]

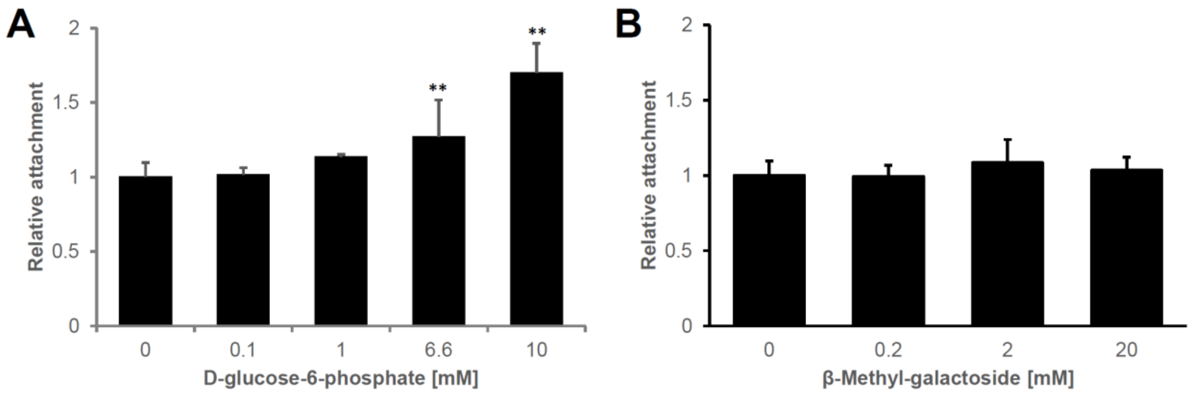

Figure S2. Attachment by wild-type *P. aeruginosa* PAO1 in response to increasing concentrations of (**A**) glucose-6-phosphate or (**B**) β-methyl galactoside. Attachment assays were carried out in LB alone or supplemented with glucose-6-phosphate or β-methyl galactoside at the concentrations indicated, with attachment assessed using CV staining. Experiments were carried out in duplicate using at least 8 technical replicates each. Error bars indicate standard deviation. **, statistical significant difference relative to LB alone ($^{**}p$-value< 0.05).
