# Supplementary material for: Glucose-6-Phosphate Acts as an Extracellular Signal of SagS To Modulate Pseudomonas aeruginosa c-di-GMP Levels, Attachment, and Biofilm Formation
Source: mSphere. 2021 Feb 10;6(1):e01231-20. doi: 10.1128/mSphere.01231-20 (PMC8544897; doi:10.1128/mSphere.01231-20)
Supplement: FIG S3 [file msphere.01231-20-sf003.pdf]

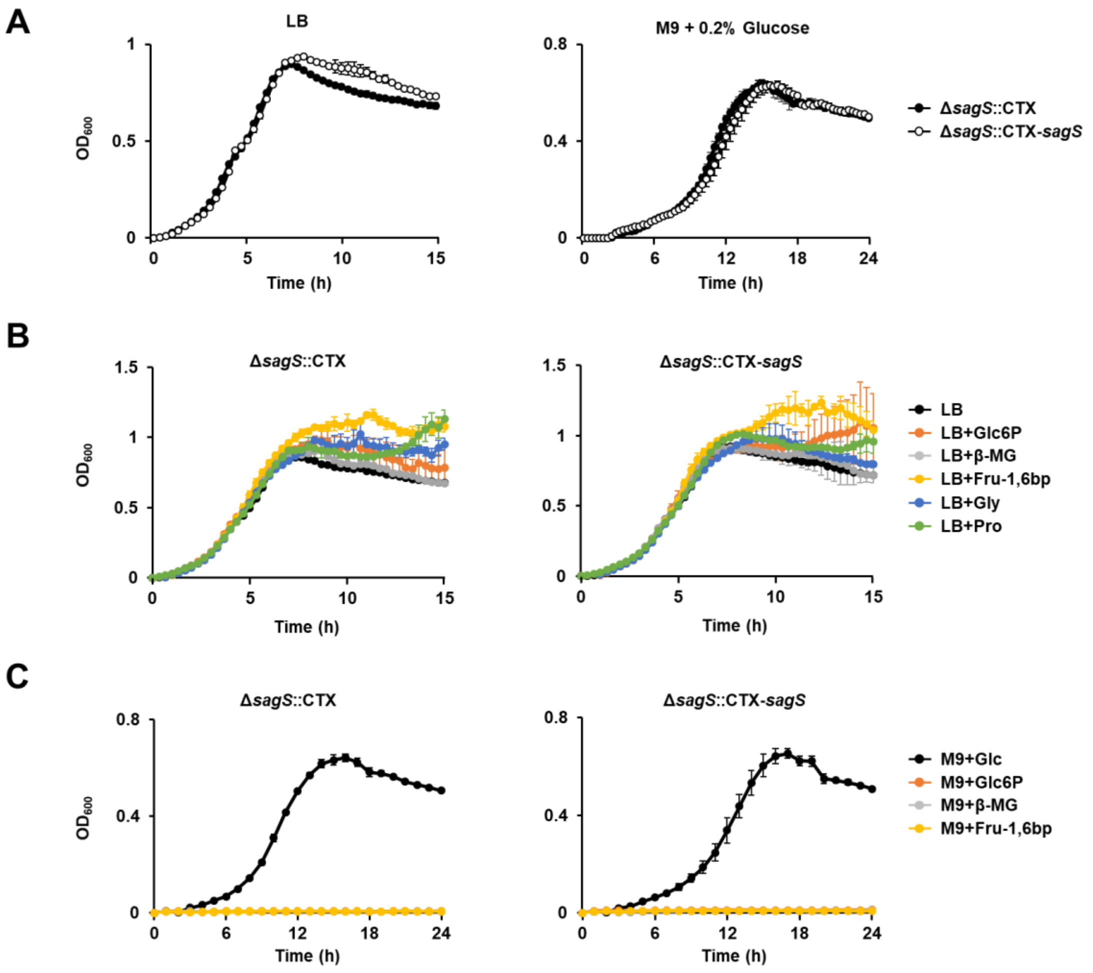

Figure S3. (**A**) Growth curves of Δ*sagS*::CTX were compared with Δ*sagS*::CTX-*sagS* in either LB medium or M9 minimal medium supplemented with 0.2% glucose (Glc) as a sole carbon source. Growth behaviors by Δ*sagS*::CTX-*sagS* and Δ*sagS*::CTX when grown (**B**) in LB medium in the absence and presence of 0.2% glucose-6-phosphate (Glc6P), β-methyl galactoside (β-MG), fructose-1,6-bisphosphate (Fur-1,6bp), glycerol (Gly) or proline (Pro), and when grown (**C**) in M9 minimal medium supplemented with the indicated compounds as the sole carbon source. After inoculation, 200 µL aliquots of each strain were transferred into a 96-well plate, and growth was monitored by measuring the absorbance at 600 nm at 37°C in a multimode microplate reader (SpectraMax i3x plate reader, Molecular Devices). The mean and standard deviation of four measurements are shown.
