# Supplementary material for: Glucose-6-Phosphate Acts as an Extracellular Signal of SagS To Modulate Pseudomonas aeruginosa c-di-GMP Levels, Attachment, and Biofilm Formation
Source: mSphere. 2021 Feb 10;6(1):e01231-20. doi: 10.1128/mSphere.01231-20 (PMC8544897; doi:10.1128/mSphere.01231-20)
Supplement: FIG S4 [file msphere.01231-20-sf004.pdf]

P. aeruginosa

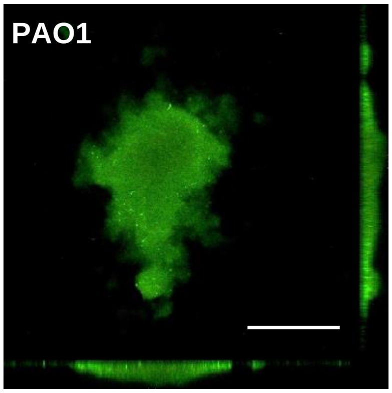

Figure S4. Representative confocal microscopy image of the biofilm architecture by *P. aeruginosa* PAO1. Biofilms were grown for 3 days in 5-fold diluted LB medium and stained prior to microscopy using the Live/Dead *Bac*Light viability stain. Bars, 100 µm.
