# Supplementary material for: Glucose-6-Phosphate Acts as an Extracellular Signal of SagS To Modulate Pseudomonas aeruginosa c-di-GMP Levels, Attachment, and Biofilm Formation
Source: mSphere. 2021 Feb 10;6(1):e01231-20. doi: 10.1128/mSphere.01231-20 (PMC8544897; doi:10.1128/mSphere.01231-20)
Supplement: TABLE S1 [file msphere.01231-20-st001.pdf]

Table S1. Strains and plasmids used in this study

Strains or plasmids	Genotypes and/or descriptions[a]	Reference or source
**Strains**		
P. aeruginosa		
PAO1	Wild-type PAO1	B.H. Holloway
Δ*sagS*	PAO1 Δ*sagS* (PA2824)	(1)
Δ*sagS*::CTX	Δ*sagS* harboring the empty pMini CTX vector, Tet^R	(2)
Δ*sagS*::CTX-*sagS*	Δ*sagS* harboring chromosomal insertion of *sagS* under the control of the *sagS* promoter at attB site, cured pMini CTX vector	(2)
Δ*nicD*	PAO1; PA4929::ISlacZ, Tet^R	(3)
ΔPA3177	PAO1 ΔPA3177	(4)
E. coli		
DH5α	F^- ϕ80*lacZ*ΔM15 Δ(*lacZYA-argF*)U169 *recA1 endA1 hsdR17*(r_k^-, m_k^+) *phoA supE44 thi-1 gyrA96 relA1 tonA*	Life Technologies
**Plasmids**		
pJN105	Arabinose-inducible gene expression vector; pBRR-1 MCS; *araC*-P_{BAD}, Gm^R	(5)
pMJT-1	*araC*-P_{BAD} cassette of pJN105 cloned into pUCP18, Amp^R ($Carb^R$)	(6)
pJN-*sagS*	C-terminal HA-tagged *sagS* cloned into pJN105 at NheI/SacI, Gm^R	(1)
pMJT-*sagS*	C-terminal HA-tagged *sagS* cloned into pMJT1 at NheI/SacI, Amp^R ($Carb^R$)	(7)
pMJT-*nicD*-V5/6xHis	C-terminal V5/6xHis-tagged *nicD* cloned into pMJT1 at NheI/XbaI, Amp^R ($Carb^R$)	(8)
pMJT-*nicD*ΔNoTMR-V5/6xHis	C-terminal V5/6xHis-tagged *nicD* lacking the DISMED2 sensory domain, cloned into pMJT1 at NheI/SmaI, Amp^R ($Carb^R$)	(8)
pCdrA::*gfp*(ASV)	pUCP22Not-P_{cdrA}-RBS-CDS-RNase III-*gfp*(ASV)-T_0-T_1, Amp^R, Gm^R	(9)
pMF440	Broad host range plasmid for constitutive expression of mCherry, Amp^R ($Carb^R$)	Michael Franklin (Addgene plasmid #62550)

[a] Tet^R , tetracyclin-resistant; Gm^R , gentamicin-resistant; Amp^R , ampicillin-resistant; $Carb^R$, carbenicillin-resistant.

Supplementary References

1. Petrova OE, Sauer K. 2011. SagS contributes to the motile-sessile switch and acts in concert with BfiSR to enable *Pseudomonas aeruginosa* biofilm formation. J Bacteriol 193:6614-6628.

2. Dingemans J, Al-Feghali RE, Lau GW, Sauer K. 2019. Controlling chronic *Pseudomonas aeruginosa* infections by strategically interfering with the sensory function of SagS. Mol Microbiol 111:1211-1228.
3. Jacobs MA, Alwood A, Thaipisuttikul I, Spencer D, Haugen E, Ernst S, Will O, Kaul R, Raymond C, Levy R, Chun-Rong L, Guenthner D, Bovee D, Olson MV, Manoil C. 2003. Comprehensive transposon mutant library of *Pseudomonas aeruginosa*. Proc Natl Acad Sci U S A 100:14339-14344.
4. Poudyal B, Sauer K. 2018. The PA3177 Gene encodes an active diguanylate cyclase that contributes to biofilm antimicrobial tolerance but not biofilm formation by *Pseudomonas aeruginosa*. Antimicrob Agents Chemother 62:e01049-18.
5. Newman JR, Fuqua C. 1999. Broad-host-range expression vectors that carry the L-arabinose-inducible *Escherichia coli araBAD* promoter and the araC regulator. Gene 227:197-203.
6. Kaneko Y, Thoendel M, Olakanmi O, Britigan BE, Singh PK. 2007. The transition metal gallium disrupts *Pseudomonas aeruginosa* iron metabolism and has antimicrobial and antibiofilm activity. J Clin Invest 117:877-888.
7. Petrova OE, Gupta K, Liao J, Goodwine JS, Sauer K. 2017. Divide and conquer: the *Pseudomonas aeruginosa* two-component hybrid SagS enables biofilm formation and recalcitrance of biofilm cells to antimicrobial agents via distinct regulatory circuits. Environ Microbiol 19:2005-2024.
8. Basu Roy A, Sauer K. 2014. Diguanylate cyclase NicD-based signalling mechanism of nutrient-induced dispersion by *Pseudomonas aeruginosa*. Mol Microbiol 94:771-793.
9. Rybtke MT, Borlee BR, Murakami K, Irie Y, Hentzer M, Nielsen TE, Givskov M, Parsek MR, Tolker-Nielsen T. 2012. Fluorescence-based reporter for gauging cyclic di-GMP levels in *Pseudomonas aeruginosa*. Appl Environ Microbiol 78:5060-5069.
