# Supplementary material for: Glucose-6-Phosphate Acts as an Extracellular Signal of SagS To Modulate Pseudomonas aeruginosa c-di-GMP Levels, Attachment, and Biofilm Formation
Source: mSphere. 2021 Feb 10;6(1):e01231-20. doi: 10.1128/mSphere.01231-20 (PMC8544897; doi:10.1128/mSphere.01231-20)
Supplement: TABLE S2 [file msphere.01231-20-st002.pdf]

Table S2. Oligonucleotides used in this study

Name	Oligonucleotide sequence (5'–3')	Use(s)
pJN105 MCS _F	TAGCGGATCCTACCTGACGC	PCR/sequencing
pJN105 MCS_R	CCATTCGCCATTCAGGCTG	
pMJT1 MCS _F	GACCGCGAATGGTGAG	
pMJT1 MCS _R	GAGCTGATACCGCTCG	
GFP-89F	GTCAGTGGAGAGGGTGAAGG	
GFP-538R	CTGCTAGTTGAACGCTTCCATC	
mCherry-F	GCGCTTCAAGGTGCACATGGAGGGC	
mCherry-R	CTTGTACAGCTCGTCCATGCCGCCG	
pelA-F	GGTGCTGGAGGACTTCATC	qRT-PCR
pelA-R	GGATGGCTGAAGGTATGGC	
pslG-F	CACGTAAGGGACTCTATCTGG	
pslG-R	CGGTCGATCTGCTTGTTGTAAC	
mexA-F	GGATCGTGACCCTGGAAGCGC	
mexA-R	GTGGCGGGGTCGATCTGGTAG	
brlR-F	CAGCGTGGTGGGCATGGAATACTT	
brlR-R	AAGCCGGCGACGTAGTGGAATTC	
nicD-F	TCACTGGGTATTGCCTATCGTCGG	
nicD-R	GGCCAGTAGCAGGAAGATGACC	
mreB-F	CATCAACAAGGTCCACGAGAAC	
mreB-R	GCTCTTCGATCAGGAACACC	
